# Mutations improving production and secretion of extracellular lipase by *Burkholderia glumae* PG1

**DOI:** 10.1007/s00253-015-7041-z

**Published:** 2015-10-17

**Authors:** Andreas Knapp, Sonja Voget, Rong Gao, Nestor Zaburannyi, Dagmar Krysciak, Michael Breuer, Bernhard Hauer, Wolfgang R. Streit, Rolf Müller, Rolf Daniel, Karl-Erich Jaeger

**Affiliations:** Institute of Molecular Enzyme Technology, Heinrich-Heine-University Düsseldorf, Düsseldorf, Germany; Institute of Microbiology and Genetics, Department of Genomic and Applied Microbiology and Göttingen Genomics Laboratory, Georg-August University Göttingen, Göttingen, Germany; Biocenter Klein Flottbek, Department of Microbiology and Biotechnology, University of Hamburg, Hamburg, Germany; Helmholtz Institute for Pharmaceutical Research Saarland, Helmholtz Centre for Infection Research and Pharmaceutical Biotechnology, Saarland University, Saarbrücken, Germany; BASF SE, Biocatalysis and Fine Chemicals Research, Ludwigshafen, Germany; Forschungszentrum Jülich GmbH, Institute of Bio- and Geosciences IBG-1: Biotechnology, Jülich, Germany; Institute of Technical Biochemistry, University of Stuttgart, Stuttgart, Germany

**Keywords:** *Burkholderia glumae*,, Lipase,, Secretion,, Enzyme production

## Abstract

*Burkholderia glumae* is a Gram-negative phytopathogenic bacterium known as the causative agent of rice panicle blight. Strain *B. glumae* PG1 is used for the production of a biotechnologically relevant lipase, which is secreted into the culture supernatant via a type II secretion pathway. We have comparatively analyzed the genome sequences of *B. glumae* PG1 wild type and a lipase overproducing strain obtained by classical strain mutagenesis. Among a total number of 72 single nucleotide polymorphisms (SNPs) identified in the genome of the production strain, two were localized in front of the *lipAB* operon and were analyzed in detail. Both mutations contribute to a 100-fold overproduction of extracellular lipase in *B. glumae* PG1 by affecting transcription of the *lipAB* operon and efficiency of lipase secretion. We analyzed each of the two SNPs separately and observed a stronger influence of the promoter mutation than of the signal peptide modification but also a cumulative effect of both mutations. Furthermore, fusion of the mutated LipA signal peptide resulted in a 2-fold increase in secretion of the heterologous reporter alkaline phosphatase from *Escherichia coli*.

**Electronic supplementary material:**

The online version of this article (doi:10.1007/s00253-015-7041-z) contains supplementary material, which is available to authorized users.

## Introduction

*Burkholderia glumae* (formerly known as *Pseudomonas glumae*) belongs to the genus *Burkholderia* within the subphylum of the β-proteobacteria (Yabuuchi et al. 1992). *B. glumae* is a moderate rice pathogen (Ham et al. 2011), which also affects several other plants (Jeong et al. 2003). Until now, just a single case of *B. glumae* isolated from an immunodeficient patient was reported (Weinberg et al. 2007). All *B. glumae* strains studied so far infect rice panicles and produce a phytotoxin called toxoflavin (Jung et al. 2011; Kim et al. 2004; Suzuki et al. 2004; Vial et al. 2007), whose production is regulated by a LuxR-LuxI-type quorum sensing (QS) system (Chun et al. 2009; Chung et al. 2011; Goo et al. 2010; Kang et al. 2008; Kim et al. 2012; Kim et al. 2007). Biotechnological applications of *Burkholderia* species mainly comprise their use as biofertilizers or bioremediation agents (Chiarini et al. 2006; Paganin et al. 2011; Suarez-Moreno et al. 2012). Furthermore, the production of an extracellular lipase (Boekema et al. 2007; Santambrogio et al. 2013) and of rhamnolipid biosurfactants (Costa et al. 2011) were described, but detailed studies regarding further biotechnological applications are missing.

Lipases represent the third largest group within the worldwide enzyme market, which is estimated to increase with a 6.3 % rate per year to reach US$6.9 billion in 2017 (Casas-Godoy et al. 2012; Freedonia 2014; Hasan et al. 2006). The production of biotechnologically relevant lipases is a well-known feature of bacteria belonging to the genera *Pseudomonas* and *Burkholderia*. These and other microbial lipases (triacylglycerol hydrolases, EC 3.1.1.3) belong to the family of α/β hydrolases and catalyze the hydrolysis of triglycerides to glycerol and fatty acids. They are the most frequently used biocatalysts in organic chemistry (Jaeger et al. 1999; Sharma and Kanwar 2014) as they are readily available at low production costs, do not require cofactors, and usually show a broad substrate specificity and high enantioselectivity as well as high stability in non-aqueous media such as ionic liquids, supercritical fluids, and organic solvents. Under non-aqueous reaction conditions, lipases can catalyze the synthesis of various esters by esterification, interesterification, and transesterification (Aravindan et al. 2007; Gandhi et al. 2000; Gupta et al. 2004; Jaeger et al. 1999; Jaeger and Eggert 2002; Jaeger et al. 1994; Jaeger and Reetz 1998; Krishna and Karanth 2002; Nagarajan 2012; Yahya et al. 1998). Additional fields of lipase application include the production of food and feed ingredients as well as intermediates for pharmaceuticals (Casas-Godoy et al. 2012; Jaeger and Eggert 2002) and, more recently, also the production of biodiesel (Narwal and Gupta 2013; Santambrogio et al. 2013). Research over the last decades focused on the development of new methods to improve enzymes by directed evolution, rational design and computational methods (Bornscheuer et al. 2012; Drepper et al. 2006). However, efficient expression and preferably also secretion of lipases are still problematic, and many biotechnologically interesting lipases, e.g., those produced by *Pseudozyma aphidis* (formerly *Candida antarctica*) or various *Pseudomonas* species, can be produced but not efficiently secreted in *Escherichia coli* thus requiring optimization of homologous expression strains (Liu et al. 2006; Omori et al. 2005). Efficient secretion of enzymes into the culture medium is favored for most applications because it facilitates down-stream processing and lowers costs. *P. aeruginosa* is a well-studied Gram-negative bacterium for which a wealth of molecular biological and biochemical methods are available (Filloux and Ramos (Eds.) 2014), and it also produces and secretes biotechnologically relevant compounds including lipases and rhamnolipid biosurfactants (Dusane et al. 2010; Rosenau and Jaeger 2000). However, as an opportunistic human pathogen, *P. aeruginosa* will not be used for the majority of industrial applications. The company BASF SE discovered that a lipase similar to the one produced by *P. aeruginosa* is secreted by *B. glumae* PG1 and can be used to produce enantiopure alcohols and amines as intermediates in the synthesis of pharmaceuticals (Balkenhohl et al. 1997; Boekema et al. 2007). Classical mutagenesis methods were applied to construct the lipase overproducing strain *B. glumae* LU8093 derived from the PG1 wild type (R. Braatz, R. Kurth, E. Menkerl-Conen, H. Rettenmaier, T. Friedrich, and T. Subkowski, 1992, patent application WO93/00924 A1 and (Boekema et al. 2007)). Further mutagenesis resulted in strain *B. glumae* LU2023, which showed improved activity of another biotechnological relevant enzyme, a butyneol I esterase (T. Friedrich, B. Hauer, C. Nuebling, R. Stuermer, 2001, patent application WO2002018560 A2).

The extracellular lipase LipA is encoded in an operon together with a second gene *lipB* (or *lif*) encoding a lipase-specific foldase (Frenken et al. 1993a; Frenken et al. 1993b; Frenken et al. 1992). The N-terminal signal peptide of LipA mediates its transport through the inner membrane via the Sec secretion system (Frenken et al. 1992). In the periplasm, the steric chaperone LipB interacts with the lipase (Frenken et al. 1993b; Rosenau and Jaeger 2000) resulting in the conversion of the enzymatically inactive so-called “near-native” state into an active conformation (El Khattabi et al. 2000; Pauwels et al. 2012). Secretion through the outer membrane is subsequently achieved via the type II secretion system formed by the so-called “secreton” (or “main terminal branch” of the general secretory pathway) (Filloux 2004).

In this study, two mutations in the lipase overproducing strain *B. glumae* LU8093, one inside and one in front of the lipase operon *lipAB*, were studied in detail to unravel their contribution to lipase overproduction. Furthermore, we demonstrated that increased secretion by the modified LipA signal peptide can be transferred to the secretion of the reporter enzyme PhoA.

## Material and methods

### Bacterial strains and growth conditions

*E. coli* strains DH5α (Grant et al. 1990) and S17-1 (Simon et al. 1983) were cultivated in LB medium (Carl Roth, Karlsruhe, Germany) at 37 °C. *B. glumae* LU8093 ((Balkenhohl et al. 1997) and R. Braatz, R. Kurth, E. Menkerl-Conen, H. Rettenmaier, T. Friedrich, and T. Subkowski, 1992, patent application WO 93/00924 A1), *B. glumae* PG1 wild type ((Frenken et al. 1992), and its *lipAB* deficient derivate *B. glumae* PG1Δ*lipAB* (Knorr 2010) were cultivated in LB medium at 30 °C. For analysis of lipase activities and transcript-level determination, *B. glumae* strains were cultivated for 14 h at 150 rpm. Standard cloning experiments were performed in *E. coli* DH5α. Plasmids were stabilized by using appropriate concentrations of chloramphenicol (50 μg/ml for *E. coli* and 200 μg/ml for *B. glumae*). Expression of *the lipAB* operon from plasmid pBBR-*lipAB* harboring its natural promoter was defined as native expression level. The wild type strain *B. glumae* PG1 is deposited as strain no. CBS 322.89 at the Centraalbureau voor Schimmelcultures, P.O.Box 85167, NL-3508 AD Utrecht, The Netherlands, and the closed genome sequence of *B. glumae* PG1 is deposited at the DDBJ/EMBL/GenBank under the accession CP002580 (chromosome 1) and CP002581 (chromosome 2) (Voget et al. 2015).

### Genome sequencing and SNP analysis of *B. glumae* LU8093

Genomic DNA of *B. glumae* LU8093 was isolated with the Masterpure DNA purification Kit (Epicentre, Madison, USA). Genome sequencing was carried out with a hybrid approach using the 454 GS-FLX system with Titanium chemistry (Roche Life Science, Mannheim, Germany) and the Genome Analyzer IIx (Illumina, San Diego, CA). Sequencing results in 437,363 and 3,998,786 reads, respectively. In order to identify SNPs, sequence reads of LU8093 were mapped onto the *B. glumae* PG1 reference genome (Voget et al. 2015) with the GS Reference Mapper (Roche Life Science, Mannheim, Germany). All candidate SNP positions were then manually verified by PCR-amplifying corresponding genome regions and re-sequencing these fragments. Manual editing steps were performed using the GAP4 software package v4.6 (Staden 1996).

### Recombinant DNA techniques

Standard DNA techniques were performed as described (Sambrook et al. 1989). PCR Extender System (5 Prime, Hilden, Germany) was used for amplification of DNA fragments. Other DNA-modifying enzymes were obtained from Thermo Scientific (St. Leon-Rot, Germany) using the manufacturer’s instructions. Plasmid isolation from *E. coli* DH5α was performed with innuPREP Plasmid Mini Kit (Analytic Jena, Jena, Germany). Genomic DNA from *B. glumae* PG1 (wild type) and *B. glumae* LU8093 was isolated using DNeasy® Blood & Tissue Kit (Qiagen, Hilden, Germany).

The *lipAB* wild type operon (GenBank accession number: AJK49931.1 and AJK49932.1) and the *lipAB* operon that harbors the mutations in the promoter region and the region coding for the LipA signal peptide were amplified using the isolated genomic DNAs from both strains as template and the primer pair “PG1 *lipAB* up/dn” (5′-*ATA TAT ATC TAG AAT TCA CCG GAT CGA TCG*-3′/5′-*ATA TATAAG CTTACC CGT TCG AAG CAC T*-3′). The PCR products include 249 bp upstream of the *lipA* startcodon with the predicted promoter sequence. The resulting DNA fragments harboring primer introduced restriction sites were hydrolyzed with *Xba*I and *Hind*III, and the resulting 2444-bp fragments were ligated into *Xba*I-*Hind*III-treated plasmid pBBR1-MCS (Kovach et al. 1994). The resulting plasmids were named pBBR-*lipAB* and pBBR-*lipAB*-*3*, respectively. Plasmid pBBR-*lipAB* was used as template for overlap-extension PCRs (Higuchi et al. 1988) to introduce single mutations. For the mutation in the promoter region, the primer pair “OLE PCR 1/2” (5′-*CCT GTC TAC AAT CAG ACG GCC G*-3′/5′-*CGG CCG TCT GAT TGT AGA CAG G*-3′) was used whereas the pair “OLE PCR 3/4” (5′-*GGA ACG CAT CAA TCT GAC CAT G*-3′/5′-*CAT GGT CAG ATT GAT GCG TTC C*-3′) was used for the mutation in the region coding for the signal peptide. The primer pair “PG1 *lipAB* up/dn” was used as flanking primers, and the resulting 2463 bp amplicon was then treated as described above. The resulting plasmids were named pBBR-*lipAB*-*1* (mutation in the promoter region) and pBBR-*lipAB*-*2* (mutation in the signal sequence).

### Transformation and conjugation

*E. coli* strains were transformed with plasmid DNA by heat shock transformation (Hanahan 1983). *B. glumae* strains were transformed by biparental mating with *E. coli* S17-1 as follows: For conjugation, 1 ml overnight culture of *B. glumae* was mixed with 2 ml of *E. coli* S17-1 in the exponential growth phase (O.D._580nm_ = 0.6–0.8) containing the plasmid of interest. After centrifugation (1 min, 21,000×*g*), the cell pellet was washed with 0.5 ml LB medium, resuspended in 50 μl LB medium and dropped onto a membrane filter (M24, Whatman) placed on an LB agar-plate. Cells were washed off from the filter with LB medium after 6 h at 30 °C, and the cell suspension was plated in appropriate dilutions on MME (Vogel and Bonner 1956) agar plates containing antibiotics and 0.5 % (*w*/*v*) glucose.

### Western blot analysis

Proteins from cell-free supernatants were precipitated with sodium deoxycholate and trichloroacetic acid (TCA) as described (Peterson 1977). After washing with 1/2 volume 80 % (*v*/*v*) acetone, the pellet was suspended with 2× SDS-sample puffer (50 mM Tris-HCl, 4 % (*w*/*v*) SDS, 10 % (*v*/*v*) glycerol, 10 % (*v*/*v*) 2-mercaptoethanol, 0.03 % (*w*/*v*) bromophenol blue). Proteins were separated by SDS-PAGE with a 12 % polyacrylamide gel (Laemmli 1970). Western blot analysis of LipA and LipB was performed using specific antibodies (kindly provided by Jan Tommassen, University of Utrecht, The Netherlands). A goat-anti-rabbit IgG (H + L)-HRP conjugate (BioRad, Munich, Germany) was used as secondary antibody. Specific antibody-protein interactions were detected using the ECL Western Blotting Detection system (Amersham Pharmacia, Buckinghamshire, GB) and the luminescence detector Stella (raytest, Straubenhardt, Germany).

### Lipase assay

Lipase activity in whole cell extracts and supernatants was measured with *para*-nitrophenyl palmitate (*p*-NPP) as the substrate (Winkler and Stuckmann 1979) at 410 nm in microtiter plates using a SpectraMax 250 photometer (Molecular Devices, Ismaning/München, Germany). Relative lipase activity was correlated to cell density (O.D._580nm_) and calculated as U/ml, with 1 U (unit) defined as the amount of lipase that releases 1 mmol of *para*-nitrophenol per minute (molar absorption coefficient 15 μMol^−1^ × cm^−1^).

### Transcript level determination

Two milliliters of culture were centrifuged (1 min, 21,000×*g*) and washed once with TE buffer (100 mM Tris-HCl pH 7.5, 20 mM EDTA). The cell pellet was then treated with RNeasy Mini Kit (Qiagen) according to the protocol for the isolation of bacterial RNA. DNaseI digestion was performed both, “on column” with RNase-free DNase Set (Qiagen) and after RNA elution with DNaseI (RNase-free) from Ambion® (Life Technologies, Darmstadt, Germany) according to the manufacturer’s instructions. The reverse transcription of isolated RNA into cDNA was carried out with the High Capacity cDNA Reverse Transcription Kit (Applied Biosystems™, Foster City, USA) according to the instruction manual. For subsequent real time qPCRs, 250 ng RNA were transcribed per reaction. In a separate reaction, each sample was also treated without reverse transcription to exclude DNA contaminations. The analysis of transcriptional levels of *lipA* and *lipB* was performed with real time qPCR (35 cycles) using the ΔΔCT-method (Livak and Schmittgen 2001; Schmittgen and Livak 2008). Here, the cDNA was used as template in a real time 7900HT Fast Real-Time PCR System with Power SYBR® Green PCR Master Mix (both Applied Biosystems™), and specific primers for *lipA* (5′-*CTA TCC GGT GAT CCT CGT C*-3′/5′-*GAG AGA TTC GCG ACG TAC AC*-3′), *lipB* (5′-*GTG GCA GAC GCG CTA TCA AG*-3′/5′-*CGT GAA AGT CTG CTG CCT GAG*-3′) and the constitutively expressed gene *rpoD* (5′-*GAT GAC GAC GCA ACC CAG AG*-3′/5′-*GAA CGC TTC CTT CAG CAG CA*-3′) as a reference. Primers were designed using Primer3 (Untergasser et al. 2012). The amount of PCR product was calculated as CT value by the Sequence Detection System (Version 2.3, Applied Biosystems™). PCR efficiencies were determined with the tool LinRegPCR (Ruijter et al. 2009). The CT values obtained for *lipA* and *lipB* were then related to those of the reference gene *rpoD* leading to the ΔCT value (ΔCT = CT(*gene*) − CT(*rpoD*)). By comparing the ΔCT values of a certain strain to its reference strain, the resulting ΔΔCT (ΔΔCT = ΔCT(strain) − ΔCT(reference strain)) value reflects the differences in the transcript amount of a certain gene between these two strains. Calculations were performed and statistically analyzed with REST© software (Pfaffl et al. 2002). All observed transcript exchanges are significantly different from the control sample (*p* < 0.05, calculated with REST©).

## Results

### Comparison of *B. glumae* wild type and the lipase production strain LU8093

**Fig. 1:**
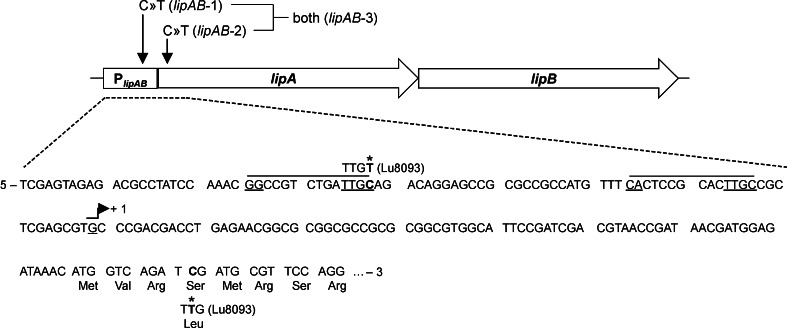
Two mutations were identified by comparative genome sequencing and localized to the *lipAB* operon of the production strain *B. glumae* LU8093. The first mutation is located in the *lipAB* promoter region (P_*lipAB*_) and is present in the constructed variant *lipAB*-1; the second mutation located in the LipA signal peptide coding sequence is present in the constructed variant *lipAB*-2; variant *lipAB*-3 contains both mutations. Two putative binding sites for δ^54^ transcription factors and the transcription start (+1) are *underlined* in the DNA sequence shown below (Beselin 2005). Coding triplets no. 1–7 of *lipA* are translated into amino acid sequence, and mutations identified in *B. glumae* LU8093 are marked with *asterisks*. The amino acid exchange resulting from mutation *lipAB*-2 is indicated

The extracellular lipase LipA produced by *B. glumae* PG1 is used by BASF SE for the production of enantiopure building blocks (Liese et al. 2006). Therefore, the lipase production strain *B. glumae* LU8093 was constructed from *B. glumae* PG1 by repeated rounds of random mutagenesis and subsequent assays for increased extracellular lipase production R. Braatz, R. Kurth, E. Menkerl-Conen, H. Rettenmaier, T. Friedrich, and T. Subkowski, 1992, patent application WO 93/00924 A1). We compared the genome sequences of *B. glumae* PG1 (Voget et al. 2015) and *B. glumae* LU8093 and identified in the production strain 72 SNPs of which 51 were located on chromosome 1, with 29 non-synonymous, 16 synonymous and six intergenic ones. From 21 SNPs found on chromosome 2, 13 were non-synonymous, five synonymous and three intergenic. Among the 72 SNPs identified in the *B. glumae* LU8093 chromosomes, two were localized within the lipase operon on chromosome 2; one in the putative promoter region and the second in the region encoding the LipA signal peptide (Fig. 1).

**Fig. 2:**
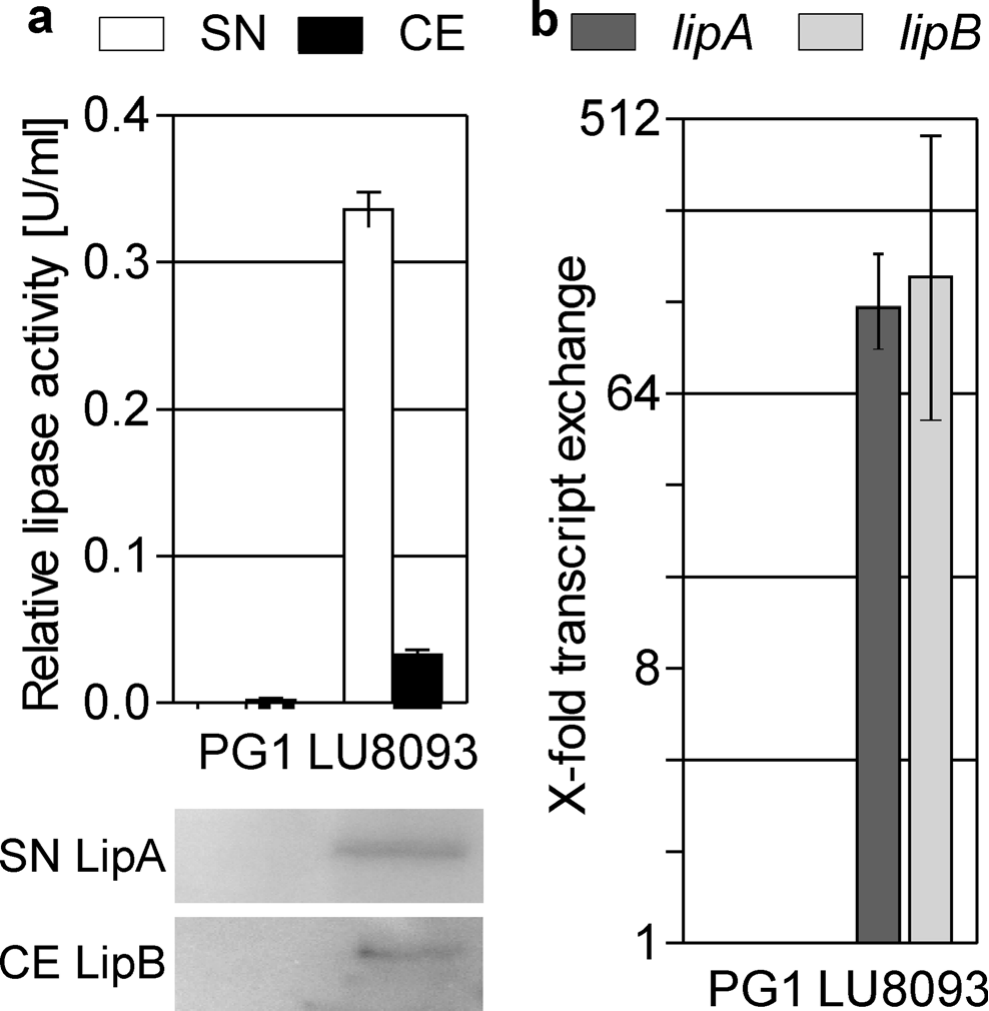
Lipase production of *B. glumae* PG1 wild type (PG1) and production strain *B. glumae* LU8093. **a** Relative lipase activity in the supernatant (SN) and cell extract (CE). LipA was detected in culture supernatants (SN LipA) and LipB in cell extract (CE LipB) by Western blotting after SDS-PAGE. Samples of 10 μl were loaded into each lane corresponding to a cell density of O.D._580nm_ = 5 for cell extracts and O.D._580nm_ = 50 for supernatants. **b** Relative change of *lipA* and *lipB* transcript levels in *B. glumae* LU8093 compared to the wild type *B. glumae* PG1 (arbitrarily set as 1). *Error bars* show standard deviations derived from examination of three biological replicates. All changes in transcript level are significant (see “Material and methods” section)

We first determined lipase activity and protein amount in cell extracts and culture supernatants obtained from *B. glumae* PG1 wild type and the production strain LU8093 demonstrating that the production strain produced more lipase than the wild type (Fig. 2a). Previous studies using fusions of the lipase promoter with GFP indicated an increased transcription rate of *lipA* in the production strain *B. glumae* LU8093 (Boekema et al. 2007). Thus, we quantified the transcription levels of *lipA* by qPCR and determined a 100-fold increase in the production strain (Fig. 2b).

### Two mutations localized within the lipase operon *lipAB* increase lipase production and secretion

**Fig. 3:**
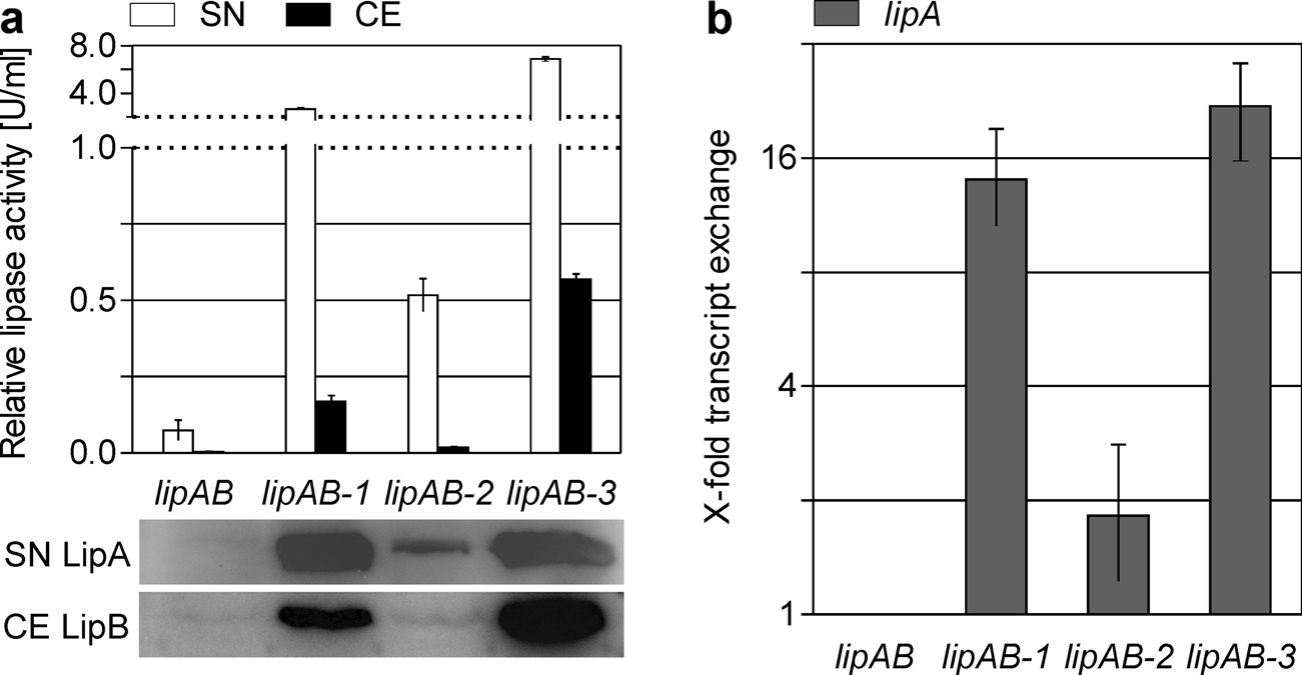
Expression of different lipase operons in *B. glumae* PG1Δ*lipAB.* **a** Relative lipase activity in cell-free supernatants (SN) and cell extracts (CE). LipA in supernatants (SN LipA) and LipB in cell extracts (CE LipB) were detected by Western blotting after SDS-PAGE with each lane containing 10 μl sample corresponding to a cell density of O.D._580nm_ = 5 for cell extracts and O.D._580nm_ = 50 for supernatants. **b** Relative change of *lipA* transcript levels in strains harboring a mutated *lipAB* operon (*lipAB-1* to −*3*) compared to the wild type operon *lipAB* (arbitrarily set as 1). *Error bars* show standard deviations derived from examination of three biological replicates. All changes in transcript level are significant (see “Material and methods” section)

The mutations identified in front of the *lipAB* operon were analyzed both separately and in combination by expression of the respective genes in a *lipAB*-deficient *B. glumae* PG1 strain (PG1Δ*lipAB*) to avoid basal expression of genome-encoded *lipAB*. To ensure that extracellular lipase activities were not caused by cell lysis, we determined cytoplasmic β-lactamase activities in cell-free culture supernatants. These activities were always less than 10 % of the overall activities for all strains tested (Fig.S1) indicating that the observed effects of the mutations on extracellular lipase levels were not caused by significant cell lysis. As shown in Fig. 3a, the mutation in the promoter region of *lipAB* (*lipAB*-*1*) resulted in a 38-fold increased lipase activity in the supernatant (~2.68 compared to ~0.07 U/ml) and 42-fold in the cell extract (~0.168 compared to ~0.004 U/ml). The mutation in the signal peptide (*lipAB*-*2*) led to a slight (~4–7-fold) increase of lipase activity in the supernatant and the cell extract, whereas the combination of both mutations (*lipAB*-*3*) resulted in ~100-fold increased activity in the supernatant (~6.87 U/ml) and ~140-fold increased activity (~0.57 U/ml) in the whole cell extract. It should be noted here that lower lipase activities of *B. glumae* PG1 wild type and *B. glumae* LU8093 as shown in Fig. 2a can be attributed to the fact that these strains harbor just one chromosomal copy of the *lipAB* operon. The increased lipolytic activity of *B. glumae* PG1Δ*lipAB* expressing plasmid-encoded lipase variants corresponded to increased production and secretion as determined by Western blot analysis of LipA in cell-free supernatants (Fig. 3a, bottom). Remarkably, a significantly increased amount of LipB was detected only in the strains harboring the promoter mutation (*lipAB*-*1* and *lipAB*-*3*).

### A mutation in the *lipAB* promoter increases the transcript level of *lipA*

Next, we analyzed the influence of these mutations on the *lipA* transcript level by qPCR (see Fig. 3b). Whereas the mutation in the signal sequence (*lipAB-2*) exhibited just a slight effect at the transcript level, the promoter mutation (*lipAB*-*1* and *lipAB*-*3*) led to an increase by a factor of 16. Interestingly, we observed that the transcript levels of *lipA* and *lipB* were differentially affected by the two mutations. While the *lipA* transcript level was increased by the promoter mutation, the amount of *lipB* transcript remained unaffected (data not shown). This may be explained by a faster degradation of the *lipB* transcript as already suggested by Frenken et al. (Frenken et al. 1993a). This assumption is further supported by the observation that more LipB was detected by Western blot analysis in strains harboring a lipase operon with the promoter mutation than in strains with the wild type operon (see Fig. 3a, bottom). Apparently, more *lipB* transcript could be produced and translated, but may be degraded faster than *lipA* transcript.

### A signal peptide mutation in LipA improves secretion in *B. glumae* PG1

The second mutation identified in the *lipAB* operon results in an exchange of serine to leucine at position 4 of the LipA signal peptide. This mutation has almost no effect on *lipA* transcription rate, but caused a remarkable increase of extracellular lipase amount (see *lipAB-2* in Fig. 3). The replacement of a polar serine by a hydrophobic leucine residue increases the hydrophobicity of the LipA signal peptide and may thus facilitate its interaction with the Sec-machinery thereby accelerating transport of LipA through the bacterial inner membrane (Driessen and Nouwen 2008). This hypothesis was tested by construction of alkaline phosphatase PhoA fusions to wild type and mutant LipA signal peptides and determination of PhoA activities in *B. glumae* PG1 and PG1Δ*lipAB*. PhoA shows enzymatic activity only after transport across the inner membrane and is therefore used as secretion reporter (Manoil et al. 1990). The LipA signal peptide derived from *B. glumae* LU8093 carrying mutation S4L resulted in a 2-fold increased PhoA activity (Fig. S2).

In summary, these results indicate that the combination of both mutations in the *lipAB* operon (see *lipAB-3* in Fig. 3) results in an increased transcription rate as well as in increased lipase secretion. In addition, we did not observe any growth defects of *B. glumae* PG1Δ*lipAB* expressing plasmid-encoded *lipAB-1* or *lipAB-2* compared to the wild type *lipAB* operon or the empty vector control, respectively. Expression of *lipAB-3* led to a slightly decreased cell density in the stationary growth phase after 24 h (O.D._580nm_ = 1.2 compared to 1.7 for wild type *lipAB*), which was, however, not observed upon comparing growth of *B. glumae* LU8093 with the wild type strain PG1 (data not shown).

## Discussion

In this study, we analyzed the increased lipase production of the industrial production strain *B. glumae* LU8093 (Fig. 2) which is a derivate of the wild type strain *B. glumae* PG1. The comparison of the genome sequences revealed 72 SNPs introduced in the production strain by classical mutagenesis methods with two of them being mainly responsible for increased lipase production and secretion (Fig. 3). One of these two mutations is located in the putative *lipA* promoter region (Fig. 1). A previous study determined the transcriptional start site located 78 bases upstream of the *lipA* start codon and the presence of two putative δ^54^-dependent promoters (Beselin 2005) with the first one located at a conserved distance of −24/−12 bp upstream of the transcriptional start (Barrios et al. 1999), and the second one in a distance of −63/−51 bp. This second putative promoter site fits perfectly with the δ^54^ consensus motif GG-N_8_-TTGC (Barrios et al. 1999). The promoter mutation analyzed in this study changes this motif from −TTGC to −TTGT (see Fig. 1). One would expect that this C-to-T transition decreases the *lipA* transcription rate, but surprisingly, it causes an increase in *lipA* transcript level. The reasons are presently unknown; however, Boekema et al. have demonstrated that lipase expression in *B. glumae* PG1 but not in LU8093 is prone to catabolite repression (Boekema et al. 2007). The promoter mutation may thus affect binding of not only δ^54^ but also of other, so far unknown, transcriptional regulators. A likely candidate could be the cAMP receptor protein (CRP) which was shown to repress the *P. putida* δ^54^-promoter *Pu* in a cAMP-dependent manner (Zhang et al. 2014). In *B. glumae* LU8093, CRP binding affinity to the mutated promoter may be diminished resulting in missing catabolic repression and correspondingly in an overall increased transcription rate. As lipase expression in *B. glumae* is also quorum sensing regulated (Devescovi et al. 2007), the promoter SNP may additionally uncouple lipase gene expression from quorum sensing regulation. The second mutation is located in the *lipA* sequence coding for the 32 amino acid long signal peptide of LipA and changes the polar residue serine with a hydropathy value of −0.8 to a more hydrophobic leucine with a hydropathy value of +3.8 (Kyte and Doolittle 1982) thereby increasing lipase secretion (Fig. 3). This observation agrees with prior studies that showed improved protein secretion by signal peptide modifications (Yoon et al. 2010). Additive effects of additional modifications, as for example observed for heterologous protein secretion in *Lactococcus lactis* (Ng and Sarkar 2013), could further increase LipA secretion in *B. glumae*. Notably, we could demonstrate that this same mutated signal peptide also resulted in a 2-fold increased secretion of PhoA (Fig. S2), a well-established secretion reporter protein (Manoil et al. 1990). Nevertheless, it should be noted that additional 70 SNPs were identified in the production strain *B. glumae* LU8093 which may also contribute to and further increase lipase production. Interestingly, none of the SNPs is located within or close to genes known to be involved in quorum sensing or lipase secretion.

The fact that *B. glumae* PG1 secretes a lipase of biotechnological interest which is indeed used in industrial applications (Balkenhohl et al. 1997; Liese et al. 2006) raises the question if this strain possesses additional features which could be interesting for biotechnological applications. A very recent study dealing with the capacity of *Burkholderia* to adapt to different environments revealed certain differences between *B. glumae* PG1 and other members of the plant pathogenic *Burkholderia* group (Seo et al. 2015). The most striking difference is the absence of the toxoflavin biosynthesis and transport gene cluster in *B. glumae* PG1. Toxoflavin is a phytotoxin and a major virulence factor for phytopathogenic *B. glumae* strains in rice (Ham et al. 2011; Jeong et al. 2003). *B. glumae* PG1 could serve as an alternative host for the production of biotechnological relevant compounds like rhamnolipids (Costa et al. 2011). We also identified 25 putative secondary metabolite clusters in the genome of *B. glumae* PG1 (see Table S1). This further underlines the biotechnological potential of *B. glumae* PG1 not only as a lipase producer, which was demonstrated in this study, but also as a prolific source for known and new secondary metabolites.

## Electronic supplementary material

ESM 1 (PDF 453 kb)
