# Supplementary material for: Mutations improving production and secretion of extracellular lipase by Burkholderia glumae PG1
Source: Appl Microbiol Biotechnol. 2015 Oct 17;100:1265–73. doi: 10.1007/s00253-015-7041-z (PMC4717159; doi:10.1007/s00253-015-7041-z)
Supplement: Supplementary file 1 — (PDF 453 kb) [file 253_2015_7041_MOESM1_ESM.pdf]

SUPPLEMENTARY MATERIAL

Article Title

Mutations improving production and secretion of extracellular lipase by *Burkholderia glumae* PG1

Journal Name: *Applied Microbiology and Biotechnology*

Andreas Knapp[1,*], Sonja Voget[2,*], Rong Gao[3], Nestor Zaburannyi[4], Dagmar Krysciak[3], Michael Breuer[5], Bernhard Hauer[5,+], Wolfgang R. Streit[3], Rolf Müller[4], Rolf Daniel[2], Karl-Erich Jaeger[1,6,#]

[1]Heinrich-Heine-University Düsseldorf, Institute of Molecular Enzyme Technology, Düsseldorf, Germany; [2]Georg-August University Göttingen, Institute of Microbiology and Genetics, Department of Genomic and Applied Microbiology and Göttingen Genomics Laboratory, Göttingen, Germany; [3]University Hamburg, Biocenter Klein Flottbek, Department of Microbiology and Biotechnology, University of Hamburg, Germany; [4]Helmholtz Institute for Pharmaceutical Research Saarland, Helmholtz Centre for Infection Research and Pharmaceutical Biotechnology at Saarland University, Saarbrücken, Germany; [5]BASF SE, Biocatalysis and Fine Chemicals Research, Ludwigshafen, Germany; [6]Forschungszentrum Jülich GmbH, Institute of Bio- and Geosciences IBG-1: Biotechnology, Jülich, Germany.

[+] present address: Institute of Technical Biochemistry, University of Stuttgart, Germany.

* Andreas Knapp and Sonja Voget contributed equally to this work.

[#] Correspondence to: Karl-Erich Jaeger, karl-erich.jaeger@fz-juelich.de

SUPPLEMENTARY MATERIALS AND METHODS

β-Lactamase activity assay. The β-lactamase activity was measured with the chromogenic substrate CENTA (Bebrone et al. 2001) dissolved in 100 mM potassium phosphate buffer (pH = 7.2) to a concentration of 150 µM. 10 µl of the substrate was combined with 150 µl cell-free supernatant or sonicated cell extract. Enzymatically degraded CENTA was continuously recorded by the absorbance at 405 nm and 30 °C.

Construction of signal peptide fusions with PhoA. *PhoA* genes fused to *B. glumae* PG1 or LU8093 signal peptide sequence, respectively, was synthesized by Life technologies (Darmstadt, Gemany) with terminal restriction sides and *Nde*I-*Hind*III hydrolyzed. Resulting fragments were used to replace *lipAB* operon in *Nde*I-*Hind*III hydrolyzed plasmid pBBR-*lipAB*. The resulting plasmids pBBR-*wtSP-phoA* and pBBR-*LUSP-phoA* encode the fusions under the control of wild-type *lipAB* promoter and include the sequence of one of the LipA signal peptides (32 amino acid residues) followed by the PhoA sequence lacking its own signal peptide (21 residues).

Alkaline phosphatase activity assay. The alkaline phosphatase activity from PhoA in cells grown under standard conditions was measured with 4-methylumbelliferylphosphate (MUP, Sigma-Aldrich, Deisenhofen, Germany) as described in (Monds et al. 2006). Cells (0.4 ml) were pelleted and resolved in 0.4 ml TSP salts (2 g/liter $(NH_2)SO_4$, 2 g/liter NaCl, and 1.65 g/liter KCl). Cells were lysed by adding 10 µl 0.1% (w/v) sodium dodecyl sulfate, 20 µl chloroform and incubation at 30°C for 10 min. Cell debris was pelleted (10,000 *x* g, 10 min) and the supernatant extracted. Subsequently, 100 µl 1 M Tris-HCl (pH 8.0) and 10 µl of 10 mM MUP (in Tris-HCl) was

added to the supernatant and incubated at 30°C for 30 min. Reaction was terminated by adding 20 µl 3 M NaOH. The cleavage of MUP to fluorescent 4-methylumbelliferone by alkaline phosphatase was detected in an *Infinite M1000 Pro* fluorescence photometer (Tecan, Männedorf, Germany) set at 360 nm excitation and 450 nm emission. Obtained relative fluorescent units (RFU) was correlated to cell density at $O.D._{580nm}$.

SUPPLEMENTARY FIGURES

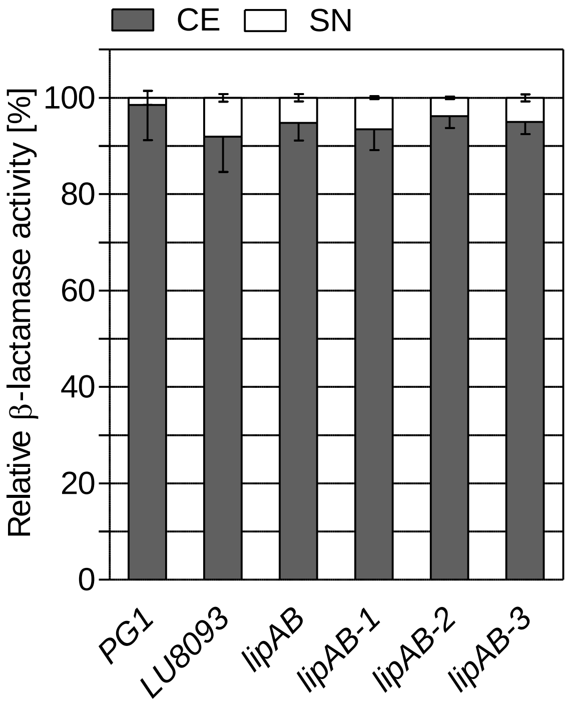

Fig. S1 Determination of relative β-lactamase activities in cell extracts (CE) and supernatants (SN) of different *B. glumae* strains as indicated at the X-axis. Lactamase activities were determined separately in cell-free supernatants and cell extracts of each culture with the chromogenic substrate CENTA. The activities in cell extract and supernatant of each strain were set as 100 % activity and each column shows the proportionate activities in the cell extract and supernatant.

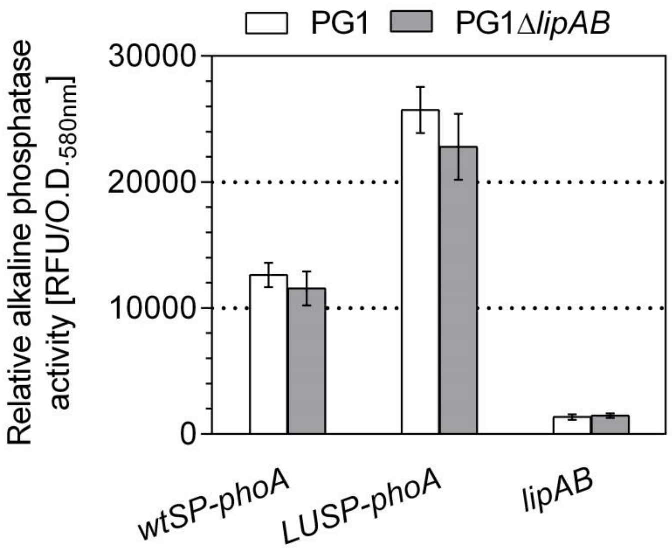

Fig. S2 Relative PhoA activity in *B. glumae* PG1 (PG1) and PG1Δ*lipAB* (PG1Δ*lipAB*) harboring different *phoA* fusion plasmids. PhoA exhibits phosphatase activity only in the periplasm and is therefore a reporter enzyme for transport across the inner membrane. The *phoA* gene is fused to the wild-type signal peptide sequence of *lipA* (*wtSP-phoA*) or the signal peptide derived from *B. glumae* LU8093 (*LUSP-phoA*), respectively. As a control, basal alkaline phosphatase activity was detected in cultures expressing plasmid-based *lipAB* (*lipAB*).

TABLE S1 Identified secondary metabolite gene clusters in *B. glumae* PG1. Cluster distribution code: B = *Burkholderia spp.*;[P] = [Polyangium]; A = *Acinetobacter spp.*; R = *Ralstonia spp.*; M = *Myxococcus spp.*; V = *Verrucosispora spp.*; P = *Pseudomonas spp.*; H = *Halothiobacillus spp.*; Mt = *Methylobacter spp.*

Cluster #	Type	Location		Key genes	Known distribution in other species
		From, bp	To, bp		
**The following clusters are from Chromosome 1:**					
**Cluster 1**	Bacteriocin	455,203	466,027	BGL_1c04090 - BGL_1c04100	
**Cluster 2**	T1pks	717,666	765,267	BGL_1c06330 - BGL_1c06490	R,B
**Cluster 3**	Hserlactone (*bgal3*)	889,059	909,637	BGL_1c07910	
**Cluster 4**	Nrps	1,344,640	1,366,545	BGL_1c11770 - BGL_1c11810	
**Cluster 5**	Bacteriocin	1,665,493	1,676,437	BGL_1c14410	B
**Cluster 6**	Nrps-t1pks	2,163,831	2,183,517	BGL_1c18980 - BGL_1c19040	B,[P]
**Cluster 7**	T1pks	2,222,887	2,270,579	BGL_1c19550 - BGL_1c19660	B
**Cluster 8**	Nrps (Pyochelin)	2,234,577	2,369,342	BGL_1c20500 - BGL_1c20630	B
**Cluster 9**	Terpene	2,749,579	2,760,412	BGL_1c23780 - BGL_1c23860	B
**Cluster 10**	Other	3,043,761	3,055,615	BGL_1c26420 - BGL_1c26510	V
**The following clusters are from Chromosome 2:**					
**Cluster 11**	Nrps-t1pks	88,975	102,059	BGL_2c00870 - BGL_2c00970	P
**Cluster 12**	Nrps-pks	103,295	133,241	BGL_2c00980 - BGL_2c01100	B
**Cluster 13**	Terpene	287,742	305,843	BGL_2c02440 - BGL_2c02630	B
**Cluster 14**	Nrps-hserlactone (*bgal2*)	457,994	507,439	BGL_2c03860 - BGL_2c03970	M
**Cluster 15**	Hserlactone (*bgal1*)	1,227,844	1,248,455	BGL_2c09850 - BGL_2c09870	A,Mt,H
**Cluster 16**	Phosphonate	1,443,527	1,479,157	BGL_2c11750 - BGL_2c11890	B
**Cluster 17**	Bacteriocin	1,852,110	1,858,332	BGL_2c14830 - BGL_2c14870	B
**Cluster 18**	Nrps-t1pks	1,974,105	2,031,300	BGL_2c15950 - BGL_2c16080	R,B,P
**Cluster 19**	Other	2,040,810	2,084,400	BGL_2c16410 - BGL_2c16520	B,P
**Cluster 20**	Nrps	2,104,675	2,187,993	BGL_2c16900 - BGL_2c17050	B
**Cluster 21**	Nrps	2,198,446	2,262,022	BGL_2c17350 - BGL_2c17480	B
**Cluster 22**	Nrps	2,256,808	2,301,211	BGL_2c17690 - BGL_2c17700	B
**Cluster 23**	Blactam	3,465,755	3,487,245	BGL_2c27580 - BGL_2c27720	
**Cluster 24**	Terpene	3,567,421	3,588,491	BGL_2c28290 - BGL_2c28460	B
**Cluster 25**	Terpene	3,712,608	3,732,771	BGL_2c29420 - BGL_2c29550	B

Comparison of certain secondary metabolite clusters (for Table S1)

We analyzed the genome for the presence of biosynthetic gene loci putatively involved in secondary metabolism and identified 25 putative clusters with antiSMASH (Blin et al. 2013). Secondary metabolites represent a versatile source of new drugs, for example antibiotics (Clardy et al. 2006). Cluster 2 shows homologies to polysaccharide gene clusters of several *Burkholderia ssp.* and especially to an ancient capsule biogenesis cluster described for the avirulent *B. thailandensis* (Yu et al. 2006). Cluster 5 shows homology to bacteriocin gene clusters from other *Burkholderia* species and cluster 6 represents a NRPS/PKS hybrid showing significant homology to those involved in the formation of natural products of the glidobactin and syringolin proteasome inhibitor families (Ramel et al. 2009; Schellenberg et al. 2007) leading to the speculation that *B. glumae* PG1 can produce such compounds as well. Cluster 8 should be regarded as NRPS cluster showing high similarity in gene structure and arrangement to many species and can be expected to be involved in the formation of an iron chelating compound with similarity to pyochelin produced by *Pseudomonas* sp. (Reimmann et al. 2001). Analysis of cluster 9 reveals a terpene biosynthetic gene locus with close homology to many other *Burkholderia* species. Cluster 18 represents a mixed NRPS/PKS gene cluster which is highly conserved among the genera *Ralstonia* and *Pseudomonas*. Cluster 19 is an atypical NRPS cluster not containing any condensation domain. It is conserved among several *Burkholderia cenocepacia* strains and can also be found in pseudomonads, although with slightly different organization of genes. Clusters 20 and 21 are other NRPS type gene clusters, both highly conserved in *B. glumae* BGR1 and cluster 21 also in *B. gladioli* BSR3. Cluster 23 represents β-lactam type gene loci without any homologs in the database. Clusters 24 and 25 represent

additional terpene synthase containing gene loci and are again highly conserved between the Bptm group and phytopathogenic *Burkholderia* species.

SUPPLEMENTARY REFERENCES

Bebrone C, Moali C, Mahy F, Rival S, Docquier JD, Rossolini GM, Fastrez J, Pratt RF, Frere JM, Galleni M (2001) CENTA as a chromogenic substrate for studying beta-lactamases. Antimicrob Agents Ch 45:1868-1871 doi:10.1128/AAC.45.6.1868-1871.2001

Blin K, Medema MH, Kazempour D, Fischbach MA, Breitling R, Takano E, Weber T (2013) antiSMASH 2.0 - a versatile platform for genome mining of secondary metabolite producers. Nucleic Acids Res 41:W204-212 doi:10.1093/nar/gkt449

Clardy J, Fischbach MA, Walsh CT (2006) New antibiotics from bacterial natural products. Nat Biotechnol 24:1541-1550 doi:10.1038/nbt1266

Monds RD, Newell PD, Schwartzman JA, O'Toole GA (2006) Conservation of the Pho regulon in *Pseudomonas fluorescens* Pf0-1. Appl Environ Microbiol 72:1910-1924 doi:10.1128/AEM.72.3.1910-1924.2006

Ramel C, Tobler M, Meyer M, Bigler L, Ebert MO, Schellenberg B, Dudler R (2009) Biosynthesis of the proteasome inhibitor syringolin A: the ureido group joining two amino acids originates from bicarbonate. BMC Biochem 10:26 doi:10.1186/1471-2091-10-26

Reimmann C, Patel HM, Serino L, Barone M, Walsh CT, Haas D (2001) Essential PchG-dependent reduction in pyochelin biosynthesis of *Pseudomonas aeruginosa*. J Bacteriol 183:813-820 doi:10.1128/JB.183.3.813-820.2001

Schellenberg B, Bigler L, Dudler R (2007) Identification of genes involved in the biosynthesis of the cytotoxic compound glidobactin from a soil bacterium. Environmental microbiology 9:1640-1650 doi:10.1111/j.1462-2920.2007.01278.x

Yu Y, Kim HS, Chua HH, Lin CH, Sim SH, Lin D, Derr A, Engels R, DeShazer D, Birren B, Nierman WC, Tan P (2006) Genomic patterns of pathogen evolution revealed by comparison of *Burkholderia pseudomallei*, the causative agent of melioidosis, to avirulent *Burkholderia thailandensis*. BMC Microbiol 6:46 doi:10.1186/1471-2180-6-46
